# Non-Auditory Participant Characteristics Influence Digit-Perception Performance: Evidence From Tasks Varying in Masker Type and Spatial Cues

**DOI:** 10.1177/23312165261468222

**Published:** 2026-09-08

**Authors:** Hannah Guest, Antje Heinrich, Kevin J. Munro, Garreth Prendergast, Rebecca E. Millman, Karolina Kluk, Christopher J. Plack

**Affiliations:** 1 Manchester Centre for Audiology and Deafness, School of Health Sciences, 5292 The University of Manchester, Manchester, UK

**Keywords:** digits in noise, spatial selective attention, speech in noise, speech perception

## Abstract

Digit-perception tasks are widely used to assess auditory function in clinical and research settings, but performance may also be shaped by non-auditory participant characteristics. This study examined whether educational attainment, forward digit span, socioeconomic deprivation, and musical experience predicted performance on three digit-perception tasks that differed in masker type and spatial demands: digits in speech-shaped noise (SSN), digits in spatially separated babble, and a spatial-selective-attention task in which digit streams were separated by 15°. Participants were 220 young adults (aged 16-17 years at baseline) with bilaterally normal audiometric thresholds, each tested twice. The narrow age range and normal hearing constrained variation in peripheral auditory function, increasing sensitivity to non-auditory influences. Linear mixed-effects models were used to examine single-predictor and multivariate associations between the predictors and each outcome. Digits-in-SSN performance was unrelated to all predictors. Digits-in-babble thresholds showed modest associations with deprivation and educational attainment in single-predictor models, but neither survived multivariate adjustment. By contrast, spatial-selective-attention performance showed robust independent associations with educational attainment, deprivation, and musical experience after mutual adjustment. Forward digit span was not a significant predictor of any task. These findings indicate that spatially demanding digit-perception tasks are vulnerable to non-auditory confounds, and that controlling for short-term memory may be insufficient to address this problem. A secondary analysis examined whether the association between musical experience and spatial selective attention might reflect confounding rather than auditory training. The musician advantage was attenuated by approximately one third after adjustment for sociodemographic factors, consistent with a substantial contribution of confounding.

## Introduction

Over recent decades, auditory research has employed a variety of digit-based tasks to assess speech perception in the presence of masking sounds. Often, the goal is to detect or characterise auditory pathology. For example, digits in speech-shaped noise have been used for hearing-loss screening (e.g., De Sousa et al., 2022), and spatially demanding digit tasks have been used to probe temporal processing deficits (e.g., Bharadwaj et al., 2015; Ruggles & Shinn-Cunningham, 2011). Such tasks are appealing because they are relatively brief, standardisable, and readily administered in the clinic or laboratory.

However, performance on any speech-perception task may be shaped by more than auditory function. Potential non-auditory influences must be considered carefully, both when designing tasks and interpreting results. This is particularly true in cross sectional studies, where observed associations risk conflating auditory pathology with extraneous participant characteristics. Chief among the non-auditory influences are cognitive factors. Multiple cognitive processes are thought to contribute to SiN perception (Rönnberg et al., 2022), with the magnitude of each contribution and the interactions among them likely varying with specific task demands and listener characteristics (Dryden et al., 2017; Heinrich, 2020). Those with established links to SiN performance include processing speed, inhibitory control, working memory, episodic memory, and crystallised IQ (Bugannim et al., 2025; Dryden et al., 2017). Closely related is attention, which is critical for selecting relevant auditory information and suppressing distractors, and is usually conceived as a limited cognitive resource (Kahneman, 1973; Pichora-Fuller et al., 2016).

Digit-perception tasks are often regarded as relatively “pure” measures of auditory function, due to their lexical simplicity, which likely reduces cognitive demands (Dryden et al., 2017). They contain almost no semantic context, so listeners do not need to perform semantic integration or use sentence-level meaning to guess missing parts. By contrast, open-set speech or sentences can trigger higher-level linguistic processing (syntax, semantics, plausibility checks) that recruit language comprehension and world knowledge (Mattys et al., 2012). Digits are drawn from a closed set of very limited response options, so a listener’s lexical search and selection is greatly simplified (Clopper et al., 2006). Short digit sequences also impose fewer demands on memory than multiword targets (Rönnberg et al., 2022). For these reasons, digits are commonly preferred when the goal is to assess auditory function (Moore et al., 2019).

However, choice of target sound is just one determinant of the non-auditory influences on a SiN task. Masker type and spatial configuration can also shape which cognitive processes are required. Widely used maskers such as stationary noise, modulated noise, babble, and single-talker speech likely impose different effects on the balance of bottom-up and top-down processing (e.g., Nuesse et al., 2018; Zekveld et al., 2013). The same is true of stimuli with spatial cues or phase adjustments that aid the segregation of target and masker (Knight et al., 2023).

Accordingly, digit targets are not a panacea. Classic dichotic listening paradigms illustrate this: they may use simple digit streams but present them in task contexts that heavily tax attention and cognitive control. Performance on dichotic-digits tasks has been independently associated with both age and educational level (Fischer et al., 2017). Potential impacts of cognitive factors on test performance are therefore a source of concern (Cameron et al., 2016).

Recent decades have seen the development of more sophisticated dichotic paradigms (e.g., Bharadwaj et al., 2015; Ruggles & Shinn-Cunningham, 2011), in which concurrent digit streams are separated not by ear but by inter-aural differences in timing, level, and/or phase. These paradigms generally aim to isolate auditory function, but non-auditory influences remain a potential confound. For instance, Ruggles and Shinn-Cunningham (2011) measured reading span to rule out effects of working memory on spatial-selective-attention performance in their normal-hearing listeners. None was observed, but the researchers did not measure other potential predictors such as sociodemographic variables or alternative cognitive measures.

In short, digit targets may reduce non-auditory influences on SiN performance, but the overall extent of such influences also depends on other task features. Given the widespread use of diverse digit-perception paradigms, it is important to characterise potential confounds. Relatively few studies have examined cognitive, demographic, educational, and socioeconomic variables that may affect performance, and comparative studies spanning multiple masker types and spatial configurations are particularly scarce. Consequently, practitioners who adopt novel digit tasks have limited empirical guidance about which non-auditory factors are likely to bias performance under given listening conditions.

A further motivation for the present study concerns musical experience, which has been proposed to benefit SiN perception via long-term auditory training. Several studies report superior SiN performance among musicians (Parbery-Clark et al., 2009, 2011), and some authors have attributed this to training-related neural plasticity. However, larger-scale and more recent investigations have raised doubts about broad transfer effects from musical training to speech perception and auditory neural measures (McKay, 2021; Whiteford et al., 2025). Given that musical participation is influenced by social structures, observed differences between musicians and non-musicians could reflect selection or confounding rather than any effect of training. It may therefore be informative to examine musical experience alongside cognitive and sociodemographic factors, to see whether any advantage for musicians persists after statistical adjustment.

The present study exploits data from the Hearing In Teens Study (https://osf.io/ghd97/) to evaluate how non-auditory participant-level characteristics influence performance on digit-perception tasks that differ in masker and spatial demands. The sample (n = 220) is narrowly age-bounded (16–17 years) and otologically healthy with bilaterally normal audiometric thresholds. Hence, variation in peripheral hearing is constrained, which should increase our ability to detect and characterise influences that arise from non-auditory sources. We therefore take an explicitly exploratory approach to ask whether task performance relates to four participant-level predictors - cognition, educational attainment, socioeconomic deprivation, and musical experience - and whether these relations differ across tasks that vary in masker complexity and reliance on spatial cues.

## Material and Methods

### Participants

Participants were aged 16 or 17 years at baseline (M = 16.9 years, SD = 0.4), all with bilaterally normal otoscopic findings, tympanograms, and pure-tone audiometric (PTA) thresholds (≤20 dB HL at 0.25-8 kHz). A total of 259 individuals were recruited through advertisements and presentations in educational institutions in the north of England, supplemented by snowball sampling. Of these, 220 participants (123 male) met the screening criteria above and completed the baseline test session. Approximately three years later, 199 returned for the final test session; 191 (106 male) again met screening criteria and completed the full study.

### Test Sessions

Participants completed two test sessions approximately three years apart (range: 2.6 to 3.3 years), both conducted in the hearing laboratories of the Manchester Centre for Audiology and Deafness. All procedures were approved by the University of Manchester Proportionate Research Ethics Committee (reference 2021-11707-19221), and written informed consent was obtained from all participants.

The test battery was developed with input from a patient-and-public-involvement panel consisting of teenagers from the same geographic area and age range as the target recruitment population (Dawes et al., 2024). Each session lasted approximately 3 hours in total, of which around 35-40 minutes were devoted to behavioural testing (audiometry, masked digit perception, and forward digit span). Frequent rest breaks were provided to minimise fatigue and loss of motivation; the longest individual behavioural task lasted approximately 8 minutes.

Audiological screening measures (otoscopy, pure-tone audiometry, and tympanometry) were conducted bilaterally at both sessions. Three digit-perception tasks were also administered at both sessions: digits in speech-shaped noise (SSN), digits in spatially separated babble, and a spatial-selective-attention task. The latter two were measured binaurally, whereas the digits-in-SSN task was measured monaurally, typically in the right ear, occasionally in the left (e.g., due to participant preference or ear piercings). The four non-auditory predictors of interest - forward digit span, educational attainment, socioeconomic deprivation, and musical experience - were assessed at the first session only.

### Screening Measures

Tympanometry was performed using a GSI TympStar Pro middle-ear analyser with a 226-Hz probe tone. Air-conduction pure-tone audiometry was conducted bilaterally at 0.25-8 kHz using a GSI Arrow audiometer with TDH-39 supra-aural headphones and MX41 cushions, in a double-walled sound-treated booth. Procedures were carried out in accordance with the recommended methods of the British Society of Audiology (2018a, 2018b).

### Self-Report Measures

Participants completed a short self-report questionnaire (available at https://osf.io/9bf7w/files/), with a researcher present to provide clarification if required. The questionnaire collected basic demographic information (age and sex at birth) and included items used to derive measures of educational attainment, socioeconomic deprivation, and musical experience.

#### Educational Attainment

All participants were enrolled in Year 11 or Year 12 of the English education system and had therefore recently completed, or were preparing to complete, General Certificate of Secondary Education (GCSE) examinations. GCSEs are nationally standardised, externally assessed qualifications typically taken at age 15–16, with grades awarded on a numerical scale from 1 (lowest) to 9 (highest).

Participants reported their final or predicted GCSE grades for all subjects taken. Because most state schools require students to sit nine GCSEs, while a small minority of selective or fee-paying schools encourage 10 or 11, educational attainment was quantified as the sum of the participant’s best nine GCSE grades. This provided a single continuous metric of academic attainment derived from a common, standardised assessment framework.

#### Deprivation

Socioeconomic deprivation was estimated from participants’ residential address data at baseline. Earlier residential histories were not recorded, nor were address data at the final session (at age 19/20), which may reflect temporary educational living arrangements rather than the family socioeconomic environment. Each participant provided their postal sector (postcode with the final two characters removed). Postal sectors were mapped to Lower-layer Super Output Areas (LSOAs) using Office for National Statistics data (ONS, 2023), and corresponding Index of Multiple Deprivation (IMD) scores were obtained for each LSOA from government statistics (Ministry of Housing, Communities and Local Government, 2019). The resulting IMD score served as an area-level estimate of socioeconomic deprivation for each participant, with higher scores indicating greater deprivation.

#### Musical Experience

Participants were asked whether they had ever regularly played a musical instrument and, if so, for how many years. For analysis, musical experience was operationalised as a binary variable: participants were classified as musicians if they reported three or more years of regular instrumental practice, and as non-musicians otherwise. This threshold was chosen to reflect sustained musical engagement rather than brief or incidental exposure.

### Forward Digit Span

The study’s cognitive measure was auditory forward digit span. The task was administered on a laptop computer in a double-walled, sound-treated booth. Auditory stimuli were presented diotically via Sennheiser HDA200 headphones, and responses were entered using a mouse and on-screen numbered buttons. Digits were spoken by a female American English talker at a rate of one digit per second, at a level of 60 dB SPL. Digits were sampled without replacement up to a list length of nine; for list lengths exceeding nine, a single digit duplication was permitted.

Each trial began with a warning cue, followed by digit presentation and participant recall. The task comprised two practice trials followed by 14 scored trials. In the scored portion of the task, list length commenced at two, increased by one following a correct response, and decreased by one after two consecutive incorrect responses. The outcome measure was the maximum list length correctly recalled.

### Digit Perception

#### General Digit-Perception Methods

Participants were seated in a double-walled, sound-treated booth for all listening tasks, listening via Sennheiser HDA200 headphones. Digit-perception testing was conducted using the Manchester Online Speech-perception Suite (browser-based software for lab and online task delivery; Shehabi et al., 2025a). All tasks employed digit-triplet targets (three consecutive single digits sampled without replacement from 0–9), but listening conditions differed in masker type, spatial configuration, and task demands. The three tasks were presented in the same order for all participants: digits in SSN, digits in babble, and finally auditory spatial selective attention.

Digits were spoken by a female British English talker; each digit token was randomly drawn from six exemplars. Each digit triplet was preceded by a carrier phrase, “The digits…”. All stimuli were wideband, including extended-high-frequency energy up to ∼16 kHz, sampled at a rate of 44.1 kHz. The inter-digit interval varied randomly between 180 and 250 ms. Stimulus signal-to-noise ratio (SNR) was adjusted by varying the levels of both the digits and the masker, holding the overall stimulus level constant at 70 dB SPL. Where spatial information was required, spatialisation was implemented by filtering stimuli with head-related transfer functions (HRTF) from the SADIE database (Armstrong et al., 2018). Participants entered perceived digits using an on-screen number pad and a mouse; visual feedback was displayed after each trial.

#### Digits in Speech-Shaped Noise

This task provided a baseline condition: a simple digits-in-noise paradigm with an energetic masker and no spatial cues available to aid segregation. The masker was speech-spectrum-shaped Gaussian noise matched to the long-term spectrum of the digit stimuli. Noise segments varied from trial to trial, sampled randomly from a 60-second soundfile. Target and masker were presented monaurally. Thresholds were measured adaptively using a 2-down/1-up staircase. Trial correctness was scored as two out of three digits correct. The adaptive track commenced at an SNR of 2 dB, with two initial reversals using 6-dB steps followed by six threshold reversals using 2-dB steps. Threshold was defined as the arithmetic mean of the SNRs at the final six reversals. Testing was preceded by a practice block in which stimuli were presented diotically but otherwise the procedure matched testing.

#### Digits in Babble

This task was designed to include two features resembling attributes of real-world noisy listening environments: a multi-talker babble and spatial separation between target and masker. The masker comprised two spatially separated streams of 10-talker female babble, presented at azimuths of -45° and +45°. On each trial, the segment for each masker stream was drawn independently at random from a 60-second soundfile, so that the two streams were uncorrelated with one another and varied across trials. Targets were presented at 0° azimuth. Threshold estimation used the same adaptive staircase as for digits-in-SSN (2-down/1-up; trial correctness = 2/3 digits correct; start SNR = 2 dB; two 6-dB initial reversals; six 2-dB threshold reversals). Threshold was the arithmetic mean of SNRs at the final six reversals.

#### Spatial Selective Attention

This task was designed to place the greatest demands on listeners’ use of spatial cues for stream selection (c.f. Ruggles & Shinn-Cunningham, 2011). On each trial a target digit triplet was presented simultaneously with two masker digit triplets. All three streams were produced by the same talker and had the same onset and sound level, such that spatial cues were the sole basis for segregation. The target was presented at 0° azimuth and the two simultaneous maskers at -15° and +15°. Performance was measured using a method of constant stimuli, rather than by adaptively varying SNR (which would introduce an additional cue to stream segregation).

Each participant completed two experimental blocks of 15 trials each, following an initial practice block (also 15 trials). To reduce the influence of block-onset effects, the first two trials of each block were discarded from analysis, leaving 13 scored triplets per block (i.e., 39 scored digits per block). To provide a robust individual metric while accommodating between-subject variability in fatigue and practice, the participant’s best block score (number of correctly recalled digits out of 39) was used as the outcome measure.

### Statistical Analysis

All analyses were conducted in R (version 4.5.0). Analysis proceeded in three stages. First, we examined bivariate associations between each of the speech-perception outcomes and each of the participant-level predictors. Second, we assessed relations among the predictors themselves to quantify interdependence and to motivate multivariate modelling. Third, we fitted multivariate mixed-effects models to estimate the independent contributions of the predictors.

The raw sum of the GCSE grades was markedly left-skewed and was normalised via a cube transformation prior to further processing; “GCSE total” below refers to the transformed variable. Forward digit span, GCSE total, and deprivation were then standardised (z-scored) prior to inclusion in the mixed-effects models; musical experience remained binary (0 = non-musician, 1 = musician). Timepoint was coded 0 = baseline and 1 = final session.

Linear mixed-effects models were fitted using the *lmer()* function from the *lmerTest* package. All models were estimated using maximum likelihood (REML = FALSE) to ensure consistency across model comparisons. Where diagnostic checks revealed heteroscedasticity, models were refit using the *lme()* function from the *nlme* package to accommodate differing residual variance across groups (see below). Correlations among predictors were computed using Spearman’s rank correlation with pairwise complete observations.

For each model, we inspected standard linear mixed-effects model assumptions: linearity of fixed effects, normality of residuals, homoscedasticity, and multicollinearity. No non-linear fixed effects or multicollinearity were observed for any model. Where residuals departed from normality, skewness and kurtosis statistics were computed (using the *moments* package) and Cook’s distances were inspected to identify influential observations. Five of the 15 models exhibited mild leptokurtosis and/or skewness. A further three showed more substantial leptokurtosis attributable to a single data point; repeating those analyses with the point excluded did not affect any conclusion, so we report the full-sample statistics. For all eight models with departures from normality, we report bootstrapped rather than Wald confidence intervals (CIs; 1000 iterations) as a precaution. Some heteroscedasticity was evident in the spatial-selective-attention models only; these were refit using *lme* with an appropriate variance structure (*varIdent*, *varPower*, or *varExp*, selected by likelihood-ratio test), without any change to substantive conclusions.

Because this work is exploratory and involves many possible comparisons, *p*-values are reported without formal multiplicity correction. Results should be regarded as hypothesis-generating, not hypothesis-confirming. The de-identified data will be made available via the Open Science Framework (https://osf.io/9bf7w/files/) to enable alternative analytic approaches.

#### Single-Predictor Associations

We initially examined digit-perception performance across both test sessions using a set of exploratory linear mixed-effects models. Twelve models were fit, corresponding to each combination of the three perceptual tasks and four non-auditory predictors. Each model included fixed main effects of timepoint and the predictor of interest, with participant identity included as a random intercept:

[digit-perception measure] ∼ [non-auditory predictor] + timepoint + (1|participant).

#### Associations Among Predictors

Pairwise Spearman rank-order correlations were computed between forward digit span, GCSE total, IMD score, and musical experience to characterise interdependence among the predictors. Associated *p*-values were obtained from Spearman’s rank correlation tests.

#### Multivariate Models

Multivariate linear mixed-effects models were constructed for each of the three digit-perception tasks, to estimate the independent contributions of the non-auditory predictors. Models included participant identity as a random intercept and fixed effects for timepoint and all four participant-level predictors entered simultaneously:

[digit-perception measure] ∼ digit span + educational attainment + deprivation + musical experience + timepoint + (1|participant).

## Results

### Single-Predictor Associations

**Figure 1.**
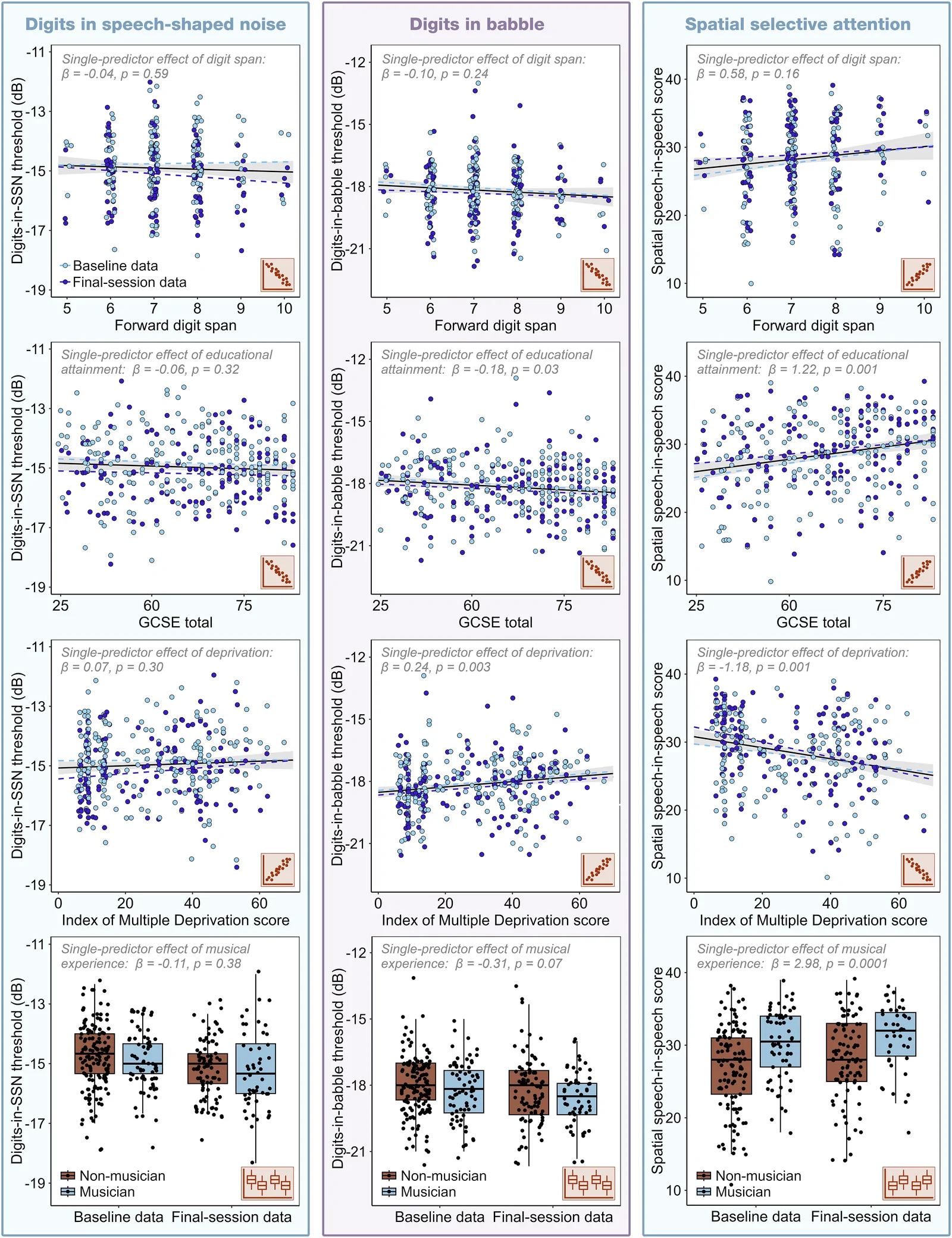
Unadjusted associations between non-auditory predictors and digit-perception performance. Each panel shows one digit-perception outcome (columns) against one predictor (rows). Each point is a single observation (each participant contributes up to two observations). In the digit-span, education, and deprivation plots, points are coloured by study timepoint (baseline or final); dashed lines show linear fits for each timepoint; the solid line shows the linear fit for the full dataset, surrounded by a shaded area indicating the 95% confidence interval. Musical experience is binary and displayed as timepoint-separated boxplots. A schematic illustration of the expected direction of each relation is presented in the lower right corner of each plot

Figure 1 plots digit-perception performance against each of the four predictors of interest.

#### Digits in Speech-Shaped Noise

There were no statistically significant associations between digits-in-SSN thresholds and deprivation, forward digit span, educational attainment, or musical experience (all *p* > 0.05).

#### Digits in Babble

Digits-in-babble thresholds were not related to forward digit span or musical experience (both *p* > 0.05). There was a weak association with educational attainment (*β* = -0.18, 95% CI [-0.33, -0.02], *p* = 0.03) and a stronger association with deprivation (higher IMD scores were associated with poorer thresholds; *β* = 0.24, 95% CI [0.08, 0.40], *p* = 0.003).

#### Spatial Selective Attention

The spatial-selective-attention measure showed robust single-predictor associations. Better performance was associated with higher educational attainment (*β* = 1.22, 95% CI [0.52, 1.96], *p* = 0.001) and musical experience (*β* = 2.98, 95% CI [1.44, 4.47], *p* = 0.0001), whereas greater deprivation was associated with poorer performance (*β* = -1.18, 95% CI [-1.91, -0.49], *p* = 0.001). By contrast, there was no significant relation to forward digit span (*p* > 0.05).

### Associations Among Predictors

**Figure 2.**
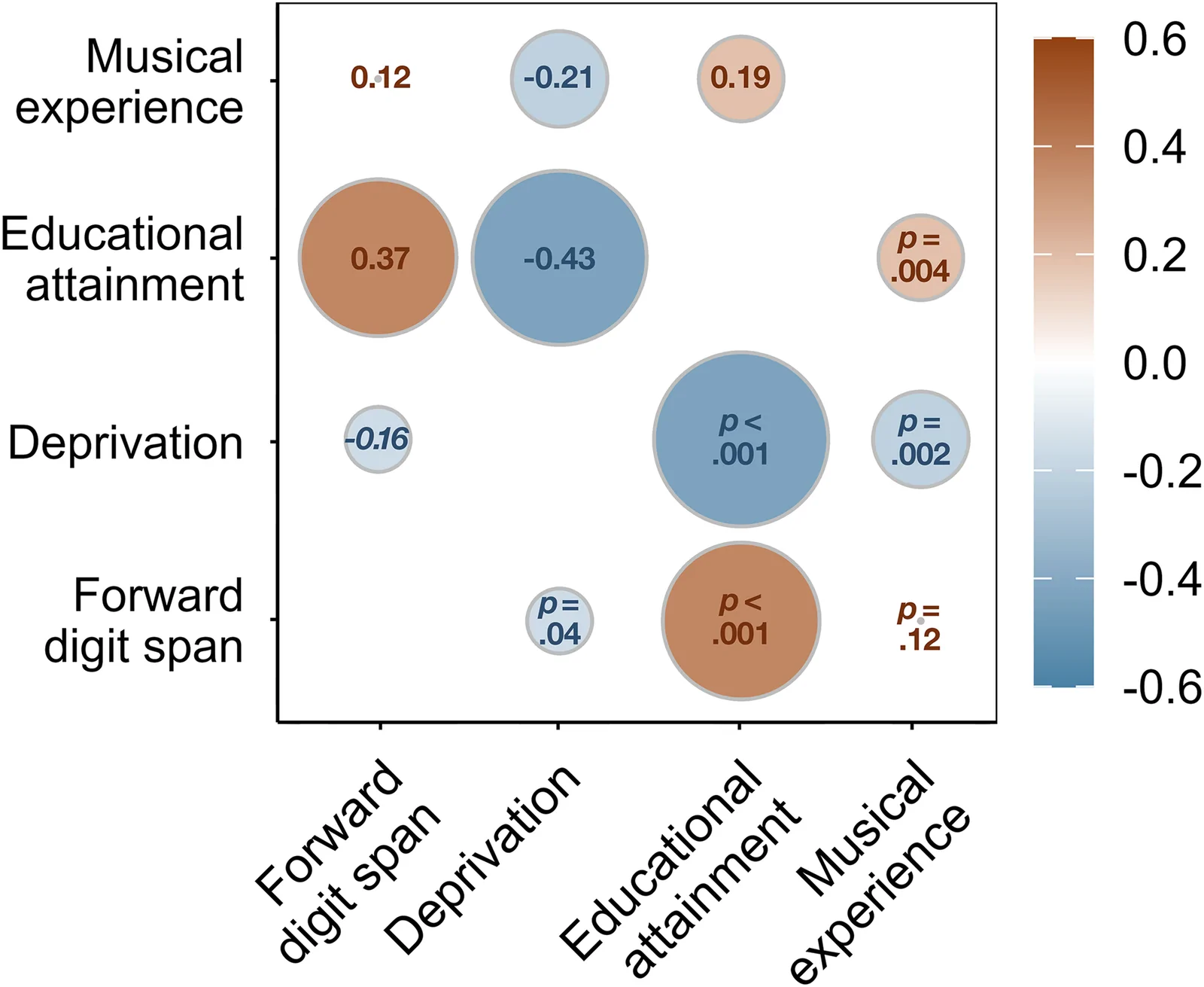
Spearman’s rank correlations among the four non-auditory predictors. Points are overlaid with Spearman’s ρ coefficients (above the diagonal) and corresponding p-values (below the diagonal). Point colour and diameter indicate the Spearman’s ρ coefficient

**Figure 3.**
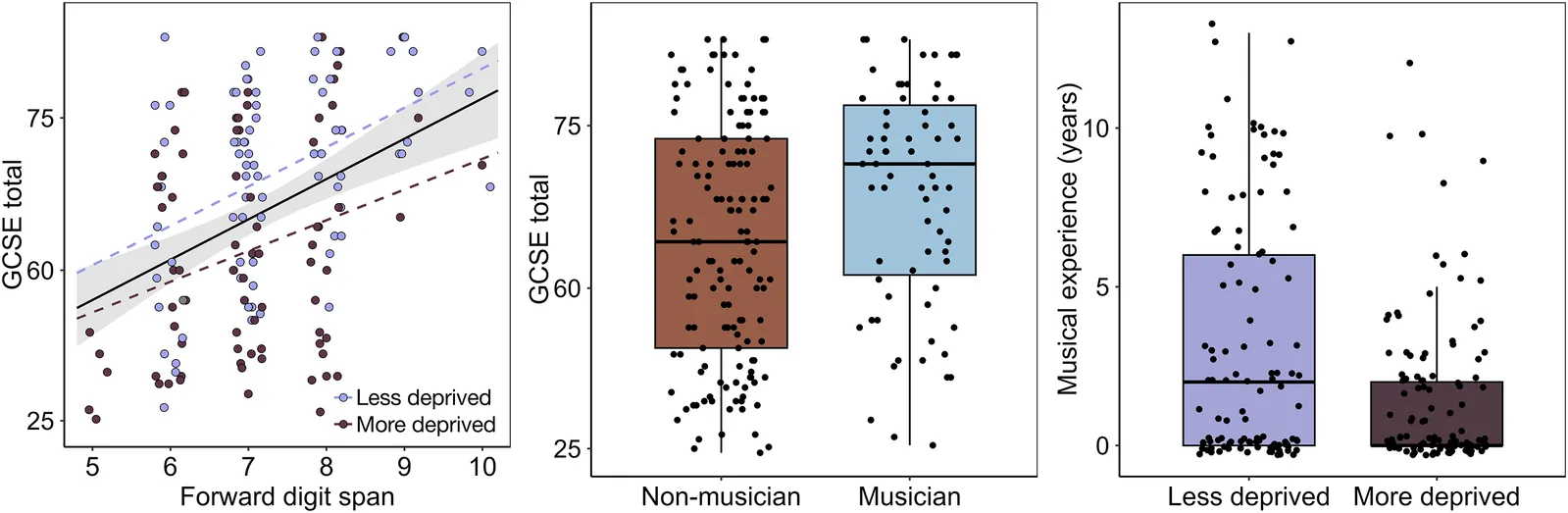
Relations among non-auditory predictors. Left panel: GCSE total (educational attainment) vs forward digit span, grouped by socioeconomic deprivation (split at the median Index of Multiple Deprivation score). Dashed lines show linear fits for each group; the solid line shows the linear fit for the full dataset, surrounded by a shaded area indicating the 95% confidence interval. Middle panel: Educational attainment (GCSE grade total) for musicians and non-musicians. Right panel: Years of musical experience for the two deprivation groups

The four non-auditory predictors were moderately intercorrelated (Figure 2), indicating that they are not independent and motivating multivariate analysis. For each of the correlations with *p* < 0.01, visual representation of the relation is presented in Figure 3.

Educational attainment (GCSE total) showed a positive association with forward digit span (*ρ* = 0.36, *p* < 0.00001), consistent with expected links between cognitive capacity and academic performance. Educational attainment was also strongly inversely related to socioeconomic deprivation (IMD score; *ρ* = −0.43, *p* < 0.00001), consistent with extensive literature (Sirin, 2005). By contrast, a much weaker association was evident between deprivation and forward digit span (*ρ* = −0.16, *p* = 0.04).

Musicians were less deprived (*ρ* = -0.21, *p* = 0.002) and more high-achieving in education than non-musicians (*ρ* = 0.19, *p* = 0.005), consistent with Corrigall and Schellenberg (2015) and Guhn et al. (2020). There was no statistically significant association between musical experience and forward digit span (*ρ* = 0.12, *p* = 0.12).

#### Multivariate Models

**Figure 4.**
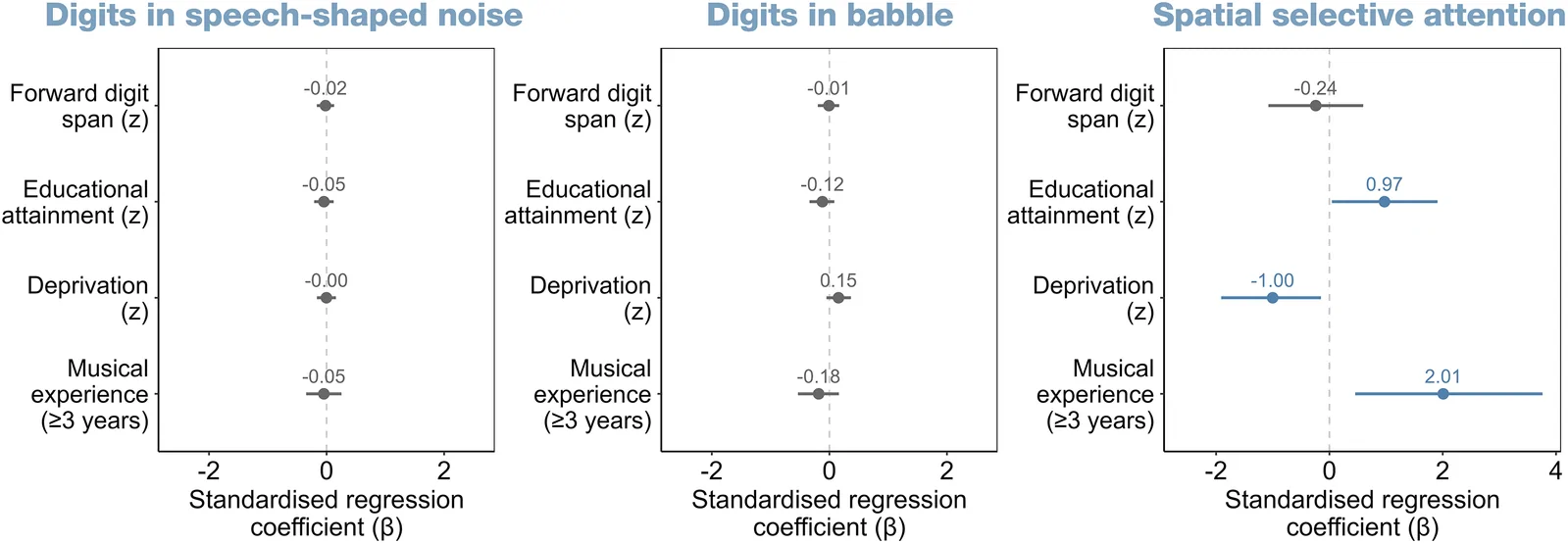
Multivariate associations between non-auditory predictors and digit-perception outcomes. Points indicate standardised regression coefficients (β); horizontal bars show 95% confidence intervals. Predictors included forward digit span, educational attainment (GCSE total), socioeconomic deprivation (IMD score), and musical experience (≥3 years). Coefficients are adjusted for all other predictors in the model. Effects not reaching statistical significance (p > 0.05) are shown in grey. Left panel: digits in speech-shaped noise. Middle panel: digits in babble. Right panel: auditory spatial selective attention

Figure 4 shows forest plots reflecting the three multivariate mixed-effects models, including regression coefficients for each of the predictors of interest (forward digit span, educational attainment, socioeconomic deprivation, and musical experience).

#### Digits in Speech-Shaped Noise

In the multivariate mixed-effects model predicting digits-in-SSN threshold, none of the four predictors of interest showed a statistically significant independent association with performance (all *p* > 0.05). Effect estimates were small and their 95% CIs included zero. Marginal and conditional R^2^ values (Nakagawa & Schielzeth, 2013) indicated that the fixed predictors explained 3% of the outcome variance; adding participant random intercepts increased total explained variance to 21% (conditional R^2^ = 0.21).

#### Digits in Babble

The full multivariate model for digits-in-babble likewise produced no independent predictors that survived simultaneous adjustment (all *p* > 0.05). Although some predictors showed nominal associations in single-predictor analyses, their effects were attenuated in the multivariate context and the CIs overlapped zero. Here, fixed effects accounted for 5% of outcome variance; including participant random intercepts raised explained variance to 30% (conditional R^2^ = 0.30).

#### Spatial Selective Attention

By contrast, the spatial-selective-attention score exhibited multiple independent associations in the multivariate analysis. Higher educational attainment predicted better performance (*β* = 0.97, 95% CI [0.05, 1.88], *p* = 0.04), greater deprivation predicted poorer performance (*β* = −1.00, 95% CI [−1.88, −0.15], *p* = 0.02), and musicians outperformed non-musicians (mean difference = 2.01, 95% CI [0.35, 3.57], *p* = 0.02). Forward digit span was not a reliable predictor in this model (*p* > 0.05). Fixed effects explained 14% of the variance; including participant random intercepts increased the total explained variance to 61% (conditional R^2^ = 0.61).

To clarify how adjustment for other participant characteristics influenced the association between musical experience and spatial-selective-attention performance, results from a minimally adjusted model were compared with those from the fully adjusted model. In the minimally adjusted model (timepoint and musical experience only), musicians outperformed non-musicians (*β* = 2.98, *p* = 0.0001). In the fully adjusted model (timepoint, forward digit span, deprivation, educational attainment, and musical experience), the coefficient for musical experience was reduced to *β* = 2.01 (*p* = 0.02), representing an attenuation of approximately 33% when adjusting for demographic and cognitive covariates.

## Discussion

### Spatially Reliant Digit-Perception Tasks Are Vulnerable to the Influence of Non-Auditory Participant Characteristics

Performance of our young, normal-hearing cohort on the simplest task (digits in SSN) was unrelated to deprivation, forward digit span, educational attainment, or musical experience, even in single-predictor analysis. Performance on a more complex task with a babble masker and spatial cues showed modest associations with deprivation and educational attainment in single-predictor analysis, but none of the predictors retained a significant independent effect in the multivariate model. By contrast, performance on the spatially reliant third task was strongly associated with deprivation, musical experience, and educational attainment in single-predictor analysis, and all three associations remained statistically significant after mutual adjustment.

The absence of non-auditory influences on the digits-in-SSN task is consistent with the view that simpler maskers reduce the contribution of cognitive factors to task performance (Heinrich, 2020), and aligns with the findings of Heinrich et al. (2015), who tested cognitive influences across a range of SiN tasks varying in both target and masker complexity. They found that digits-in-SSN performance was not predicted by attention or working memory, even in older adults with hearing loss - precisely the group most likely to exhibit such effects. Moore et al. (2014) did observe a relationship between cognition and performance on a digits-in-SSN task, independent of age, though lack of audiometric data complicates interpretation. The present sample was younger and likely more cognitively homogeneous than those of both Moore et al. (2014) and Heinrich et al. (2015), which may have limited the detectability of cognitive effects on digits-in-SSN performance.

Regarding the digits-in-babble condition, there is little directly relevant literature. To our knowledge, no prior studies have specifically examined cognitive influences on digits-in-babble performance, though a meta-analysis by Dryden et al. (2017) suggested more broadly that word recognition in background babble engages working memory. Wilson et al. (2006) examined the effects of age and hearing loss on digits-in-babble thresholds in adults but did not assess cognitive or sociodemographic influences. Moore et al. (2019) measured paediatric thresholds for digits in four-talker babble, with and without spatial separation of target and masker, and found clear maturation effects up to the mid-teens, but did not examine the cognitive processes underlying those developmental changes. The authors noted that a babble masker, relative to conventional noise, might introduce additional performance variability through informational masking, potentially reducing sensitivity to hearing loss.

In relation to our third task, several prior studies have employed digit-based spatial-selective-attention paradigms. Ruggles and Shinn-Cunningham (2011) presented target digits at 0° and masker digits at −15° and +15°, spatialised via binaural room impulse responses with added reverberation. They found no relationship between spatial-selective-attention performance and reading span, despite considerable between-listener variability in a normal-hearing sample. Subsequent work from the same laboratory using similar designs did not control for cognitive or sociodemographic variables. For example, Ruggles et al. (2012) used the same task in an expanded sample of listeners, while Bharadwaj et al. (2015) delivered two streams of digits, spatialised via simple ITDs. Theoretical justification for omitting control of cognitive factors was offered by Dai and Shinn-Cunningham (2016), who argued that narrow angles of separation (15°) between target and masker streams make such tasks primarily sensitive to the fidelity of peripheral temporal coding rather than central or cognitive factors.

In common with Ruggles and Shinn-Cunningham (2011), we found no influence of memory span on spatial-selective-attention performance. However, we found substantial independent contributions of educational attainment, socioeconomic deprivation, and musical experience. This indicates that digit-based spatial-selective-attention tasks, though designed to be sensitive to auditory deficits, are also susceptible to person-level non-auditory influences. Caution is therefore warranted in their application and interpretation, particularly in cross-sectional designs where the predictor of interest (such as occupational noise exposure) may be systematically associated with sociodemographic differences (such as educational attainment).

### Sociodemographic Factors, but Not Forward Digit Span, Predict Selective-Attention Performance

As noted above, both Ruggles and Shinn-Cunningham (2011) and the present study observed wide variation in spatial-selective-attention performance among normal-hearing listeners. In both studies, this variation was unrelated to the single cognitive measure employed (reading span in the earlier study, forward digit span in the present study). Ruggles and Shinn-Cunningham (2011) tentatively attributed the unexplained variance to peripheral temporal-coding deficits. Our findings point to an additional source: influences of non-auditory participant characteristics. Importantly, we cannot conclude that cognitive abilities play no role. Both studies relied on a single, memory-focused cognitive measure, and spatially reliant digit-perception tasks may well be sensitive to cognitive abilities that memory-span tasks do not capture.

It is therefore important to assume appropriate interpretive humility when considering the mechanisms underlying our findings - specifically, the significant independent effects of educational attainment, socioeconomic deprivation, and musical experience on spatial-selective-attention performance. Our measures of education and deprivation are necessarily coarse proxies for complex underlying circumstances, so causal inference is not straightforward. Nevertheless, it is plausible that these variables capture a broad range of traits, skills, and behaviours that support performance on challenging assessments. These may include a multitude of SiN-relevant cognitive abilities (Dryden et al., 2017), attentional resources (Pichora-Fuller et al., 2016), and valuable non-cognitive factors such as perseverance, mindset, and strategy use (Farrington et al., 2012). The development of such assets is aided by well-resourced and supportive family and school environments (Bradley & Corwyn, 2002; Farrington et al., 2012; Rakesh et al., 2025) and is predictive of educational success (Farrington et al., 2012; Zimmerman, 2002), which may explain why educational attainment and deprivation predict performance on a demanding auditory task.

These findings have practical implications for the design and interpretation of digit-perception studies, particularly those using spatially demanding tasks. They suggest that substantial variance in spatial-selective-attention performance among normal-hearing listeners can be explained by non-auditory factors, and that a single cognitive test - particularly one focused on memory span - is likely insufficient to control for these influences. Our data suggest that indexing childhood socioeconomic deprivation via address data may be a practical and informative step, given its strong associations with auditory performance and the ease with which it can be obtained. Controlling for educational attainment may also be valuable, although its feasibility will depend on the population and the availability of standardised data. Our study benefited from the fact that all participants could be characterised using a common national assessment framework (GCSEs). Whether educational adjustment would be equally effective in older or more heterogeneous populations - using measures such as years of schooling - remains an open question.

### Musical Experience May Have Fewer Direct Benefits for Listening than Supposed

A notable finding is that the association between musical experience and spatial-selective-attention performance was substantially reduced after adjustment for demographic and cognitive covariates. This observation is relevant to a broader literature reporting superior speech-in-noise performance among musicians (Parbery-Clark et al., 2009, 2011). Some authors have interpreted such associations as reflecting training-related improvements resulting from sustained musical practice. However, large-scale analyses have questioned the robustness of transfer effects from music training to speech perception and auditory neural measures (McKay, 2021; Whiteford et al., 2025).

What might underlie the unadjusted association between musical experience and listening performance? One possibility is that individuals with better innate auditory abilities are more likely to take up and persist with an instrument, and also to perform better on laboratory listening tasks - a selection effect rather than a training effect. Our data are compatible with this interpretation, since an independent association with musical experience remained even after controlling for demographic and cognitive factors. However, our results also highlight a non-auditory pathway: musical experience in our sample was associated with lower socioeconomic deprivation and higher educational attainment, and adjusting for these factors attenuated the musical-experience effect size by approximately one third. As noted in the previous subsection of the Discussion, children raised in more advantaged family and school environments develop valuable traits and skills that support performance in demanding assessments, possibly including digit-perception tasks as well as educational assessments. Importantly, it is also well established that children raised in such environments are more likely to engage in sustained music learning (e.g., Corrigall & Schellenberg, 2015).

In sum, our results do not rule out the possibility that sustained musical practice can improve certain auditory abilities. They do, however, suggest that the crude association between being a musician and outperforming peers on complex listening tasks is substantially entangled with socioeconomic and educational factors. Future work should prioritise designs and measures capable of separating selection and confounding from genuine training-related effects.

### Limitations

Several limitations should be noted. The set of non-auditory predictors examined was modest in scope, and most were coarse indicators of complex underlying constructs. Our sole cognitive measure was forward digit span, which primarily assesses short-term memory and simple attentional capacity, and does not capture the broader range of cognitive processes likely relevant to speech-in-noise perception. Socioeconomic status was estimated from area-level deprivation indices derived from residential address, rather than from individual-level income, parental occupation, or household wealth. Musical experience was operationalised as a binary variable (three or more years of regular practice versus less), sacrificing information about type of instrument, intensity of training, or current engagement. Educational attainment was quantified from examinations taken at age 15–16; the youngest 11% of participants reported predicted GCSE grades (issued mid-year by their teachers), while the remaining 89% reported final grades awarded by the examining board.

The digit-perception tasks also had limitations. The digits-in-SSN task was administered monaurally while the remaining two tasks were binaural. However, all participants had bilaterally normal pure-tone audiometry, otoscopy, and tympanometry, and given the complete absence of associations between digits-in-SSN performance and any of the predictors, it seems unlikely that binaural delivery would have materially changed these findings. Adaptive tracks were kept deliberately short to minimise the risk of fatigue and disengagement in a teenage sample - a decision informed by piloting and patient-and-public involvement - but this may have reduced the precision of individual threshold estimates. Spatial stimuli were presented using non-individualised HRTFs, potentially contributing additional between-participant variability and reducing statistical power to detect associations with the non-auditory predictors. Only three listening conditions were examined, and they differed simultaneously in masker type and spatial configuration rather than varying a single parameter at a time. Consequently, we cannot identify which specific acoustic features drive the differential sensitivity to non-auditory factors across tasks. What can be said is that the pattern of results is coherent: tasks positioned toward the complex, spatially reliant, informationally masked end of the continuum showed greater susceptibility to non-auditory influences.

One noteworthy omission is the antiphasic digits-in-noise test, which exploits interaural phase differences to support stream segregation and is widely used in remote hearing screening (De Sousa et al., 2022) due to its sensitivity to unilateral hearing loss (De Sousa et al., 2020; Shehabi et al., 2025b). Because good performance requires exploitation of interaural phase cues, the antiphasic task may plausibly engage cognitive and attentional resources to a greater degree than the standard diotic version. Given its growing role in hearing healthcare, characterising its susceptibility to non-auditory factors - and potentially incorporating such information into screening algorithms - would be a worthwhile goal for future research.

## Conclusion

Data from young, normal-hearing listeners indicate that caution is warranted in the design and interpretation of digit-perception tasks, particularly in cross-sectional research where confounding is a concern. Tasks that are heavily reliant on spatial cues appear especially vulnerable to non-auditory influences, including educational attainment, socioeconomic deprivation, and musical experience.

With respect to managing non-auditory influences, the present data provide no evidence that controlling for memory span is of value, at least in research with young, normal-hearing participants. Controlling for socioeconomic deprivation via residential address data appears worthwhile, given both its ease of measurement and its association with auditory task performance. Adjustment for educational attainment may also be informative, though the availability and appropriateness of such data will depend on the population studied.

Finally, the present data revealed a strong association between musical experience and spatial-selective-attention performance. Training-related auditory benefits cannot be ruled out, but confounding clearly plays a substantial role, because musicians tend to be more privileged and academically high-achieving than non-musicians.

## Data Availability

The anonymised study data will be made available online via the Open Science Framework (https://osf.io/9bf7w/files/) upon publication of the present article.
